# Religiosity and Mental Health: A Contribution to Understanding the Heterogeneity of Research Findings

**DOI:** 10.3390/ijerph17020494

**Published:** 2020-01-13

**Authors:** Klara Malinakova, Peter Tavel, Zdenek Meier, Jitse P. van Dijk, Sijmen A. Reijneveld

**Affiliations:** 1 Olomouc University Social Health Institute, Palacký University Olomouc, 771 11 Olomouc, Czech Republic; peter.tavel@oushi.upol.cz (P.T.); zdenek.meier@oushi.upol.cz (Z.M.); j.p.van.dijk@umcg.nl (J.P.v.D.); 2 Department of Community and Occupational Medicine, University Medical Center Groningen, University of Groningen, 9713 AV Groningen, The Netherlands; s.a.reijneveld@umcg.nl; 3 Graduate School Kosice Institute for Society and Health, P.J. Safarik University in Kosice, 040 11 Kosice, Slovakia; 4 Department of Social Medicine and Public Health, Faculty of Medicine and Dentistry, Palacký University in Olomouc, 771 47 Olomouc, Czech Republic

**Keywords:** atheism, religiosity/spirituality, mental health, attachment, measurement

## Abstract

Most studies report positive associations between religiosity and spirituality and aspects of mental health, while a small proportion report mixed or fully negative associations. The aim of this study was to assess the associations of religiosity measured more specifically, with mental health in a secular environment, using a nationally representative sample of Czech adults (*n* = 1795). We measured religious affiliation, conversion experience, non-religious attitudes and the stability of these attitudes, mental health problems, and anxiety levels. Compared to stable non-religious respondents, unstable non-religious and converted respondents who perceived God as distant were more likely to experience anxiety in close relationships, and had higher risks of worse mental health. Our findings support the idea that the heterogeneity of findings in associations between religiosity/spirituality and mental health could be due to measurement problems and variation in the degree of secularity. A shift towards religiosity could be expected to be seen in a substantial part of non-religious respondents in problematic times.

## 1. Introduction

Most studies report a positive association between religiosity and spirituality (R/S) and aspects of mental health (MH) [1], such as a higher life-satisfaction and meaning in life [2], a lower prevalence of anxiety and depression [3,4], suicidal tendencies [5] and substance abuse [6], and better cognitive functioning [7]. Some authors even present spiritual health as a fundamental dimension of people’s overall health and well-being, permeating and integrating all other dimensions of health [8]. However, a small proportion of such studies report either mixed or negative associations [1]. Understanding why these findings deviate may add to our understanding of the underlying process.

Among the possible explanations, problems with measurement [9] are most often mentioned, related to the fact that both spirituality and religiosity are hard to measure as multidimensional constructs [10]. Religiosity is most often seen as participation in an organised religion (i.e., in an organized system of beliefs and practices) [1], while spirituality can be understood in many ways, ranging from a traditional understanding of spirituality as an expression of religiosity, in search of the sacred, through to a humanistic view of spirituality devoid of religion [8]. Recently, spirituality has mostly been considered to be a more personal, subjective experience [11] that often includes a connection to the transcendent (e.g., one’s relationship to God); a connectedness to oneself, others and the world; feelings of peace, love and harmony; and the sense of an ultimate meaning of life [12]. Thus, definitions can differ to a high degree, especially regarding spirituality [9]. This hinders comparison of the various studies, because results might differ due to the definitions used. Approaches that would touch both the external and internal aspect of R/S are probably the best solution.

Another explanation of the heterogeneous findings on R/S and mental health could be a failure to take socio-cultural factors and context into account [13]. To date, most research has been performed in predominantly religious countries [14], but associations may be different for those living in more secular countries. Some studies report that religious individuals show better subjective health only in countries in which religiosity is common and socially desirable [15,16] and that having the same religion in two different cultural contexts may have opposing outcomes [17]. Hayward and Elliott [18] found R/S in secular countries to be associated with adverse health outcomes and linked explanations for this with the social norms and governmental policies in the country concerned. However, there is a lack of research that takes such individual factors into greater account.

A third explanation for some heterogeneous findings on the association of R/S and MH regards more internal factors, such as attachment styles, which also differ between various cultures [19]. Moreover, attachment style has already been associated with some dimensions of R/S. Research shows that a believer’s perceived relationship with God meets the defining criteria for attachment relationships and can function psychologically—much like other attachments [20], and that this relationship is also associated with mental [21] and physical health [22]. In line with these findings, Fisher [23] showed that relating to God also contributes to personal happiness and that it has a strong positive impact on spiritual well-being [24]. Taking into account participants’ image of God may therefore represent another way of taking into account the heterogeneous nature of religiosity and spirituality.

Kirkpatrick [25] also linked attachment theory with the process of religious conversion and found that women with an anxious attachment style were more likely to become religiously converted than women with a secure or avoidant attachment style. Other studies support these findings [26,27], which imply that religious instability may have similar roots as those of unstable emotional attachment. Religious conversion, or the stability of religious attitudes in general, could also contribute to the heterogeneity of the findings regarding R/S and MH in association with the socio-cultural context. In line with the person–environment fit model [28], which is defined as the degree to which individual and environmental characteristics match, we can assume a different conversion process in religious and secular countries. People often convert to religion in times of distress in a difficult life situation [29,30]. Nevertheless, while people might turn to religion more easily in predominantly religious countries because conversion is socially acceptable, in secular ones, the “cost of the conversion” [31] is higher. Where a difficult life situation represents the main reason for conversion, we could expect a higher level of stress (i.e., a worse psychological condition) among converts. The Czech Republic is one of the most secular countries in the world. According to some sources, it is the country with the highest percentage (76.4%) of religiously unaffiliated people in the world [32], which represents a unique setting to assess the effects of conversion.

Therefore, the aim of this study was to assess the associations of religiosity measured more specifically (i.e., as perceived closeness to God and of the stability of religious attitudes) with MH (i.e., the attachment insecurity and other mental health problems) in a secular environment.

## 2. Materials and Methods

### 2.1. Participants and Procedure

A national sample of the Czech population aged fifteen years and older was obtained using a two-step procedure. In the first step, the questionnaire and all further procedures were piloted among 206 participants. This led to the final version of the survey. In the second step, another 2184 participants were randomly chosen with the help of quota sampling and asked to participate in a study on the problematics of health, life experiences, and attitudes and lifestyle. Of these respondents, 384 (17.6%) refused to participate in the survey. Participants reported a lack of time (39.2%), a lack of interest in or distrust in research in general (24.0%), the personal nature of the questions (17.2%) and the length and difficulty of the questionnaire (11.2%) among the main reasons for refusal.

Data were collected by professionally trained administrators in September and October 2016 with a standardized interview with the respondents (face-to-face). Five questionnaires were excluded because of incomplete information regarding any of the religious questions, leading to a final sample of 1795. Participants received written information on the aim of the study and the anonymized handling of data, and were made familiar with the system. Participation in the survey was fully voluntary, so the respondents could stop responding in the survey at any time before or during the interview. Therefore, starting the survey was seen as providing informed consent. The full study design was approved by the Ethics Committee of the Olomouc University Social Health Institute, Palacký University Olomouc (No. 2016/3) and conducted in accordance with the ethical requirements as formulated by the Convention on Human Rights and Biomedicine (40/2000 Coll.).

### 2.2. Measures

Religious background and the stability of religious attitudes were assessed by the questions on religious affiliation, faith education, conversion experience, non-religious attitudes, stability of non-religious attitudes and God Image. MH was assessed by the Experiences in Close Relationships-Revised questionnaire and by the Brief Symptom Inventory (BSI-53). The translation process of all questionnaires was done according to forward–backward translation.

Religious affiliation was measured by the question: “At present, would you call yourself a believer?” with possible answers: yes, I am a member of a church or religious society; yes, but I am not a member of a church or religious society; no; no, I am a convinced atheist.

Conversion experience was assessed only among respondents who labelled themselves as believers by the following questions: (1) “Have you ever experienced something that could be called a religious conversion (acceptance or change of denomination)?” with possible answers: yes; no. The participants with the conversion experience were subsequently considered as converts. (2) For converts: “How important a role did the following factors have in your conversion?” Each factor (difficult life situation, personal religious experience, example of other people, religious literature or other) was assessed with the following possible answers: had a key role; partly contributed; had no role.

*Stability of non-religious attitudes* was assessed only among respondents who reported that they are non-believers by the following question: “What circumstances would motivate you to pray personally or to attend a religious service?” with possible multiple answers: a difficult life situation (illness, death of a close person, financial problems); psychological problems (anxiety, depression); search for the meaning of life; gratitude; politeness towards family members, friends, etc.; need for a community (desire to belong somewhere); others (please specify); nothing. Participants who chose the options “politeness” or “nothing” were further considered as stable non-religious—that is, participants who would not show any religious action of their own volition. The rest were considered as unstable non-religious—that is, participants, who, under certain circumstances, could use religion as a coping strategy or could be positively motivated to show some religious action.

Perceived closeness to God was assessed only among respondents who labelled themselves as believers, using one question from the 2005 Baylor study: “How well do you feel that the word ‘distant’ describes God?” Possible answers were the following: very well; somewhat well; not very well; not at all. Respondents who chose the option “not at all” were considered as perceiving God as close, the rest as perceiving God as distant.

**Figure 1:**
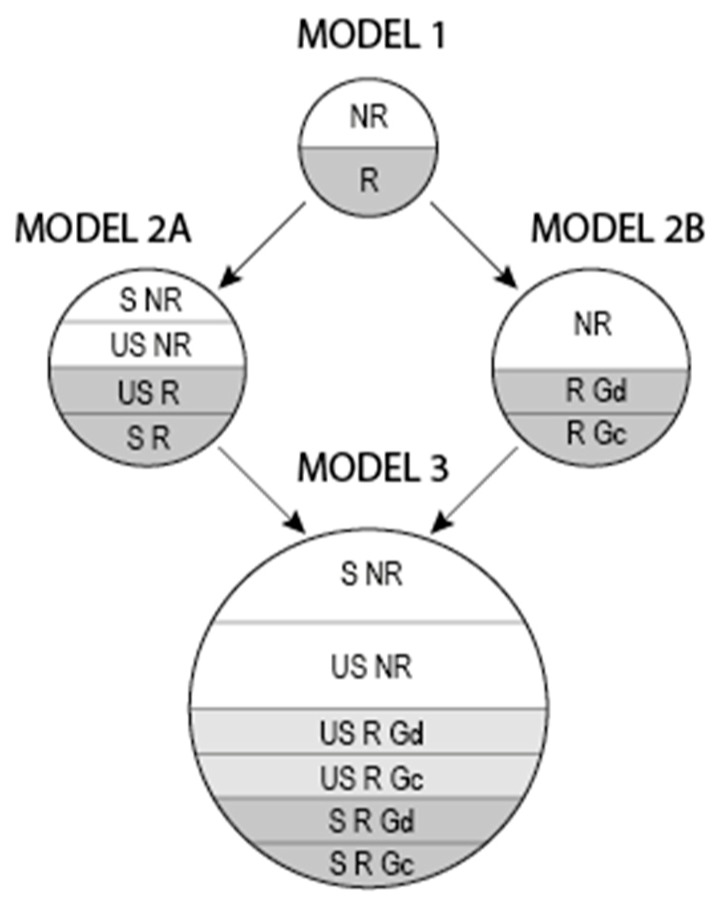
Categorisation of respondents according to religious affiliation combined with perceived closeness to God and stability of religious attitudes. NR: non-religious; R: religious; S: stable; US: unstable; Gd: perceiving God as distant; Gc: perceiving God as close.

The respondents were categorised based on the combination of religious affiliation with the other variables, to be used as independent variables in various models for the association with MH. In the first model (1), participants were dichotomised only according to their religious affiliation. In 2A, religiously affiliated participants were also categorised according to perceived closeness to God. In Model 2B, participants were categorised according to both religious affiliation and the stability of religious attitudes: stable non-religious, unstable non-religious, unstable religious (converts) and stable religious. Model 3 included the independent variables of both model 2A and 2B, resulting in six categories (see Figure 1). Thus, in all the models, the categories were clearly distinct, with no overlaps.

The dependent variable *anxiety in close relationships* was assessed using the anxiety subscale of the Experiences in Close Relationships-Revised questionnaire (ECR-R) [33]. It is composed of 18 items rated on a seven-point scale with possible answers ranging from “totally disagree” (1) to “totally agree” (7) and measures the extent to which people are uncomfortable being close to others. In the main analyses, it was assessed as a binary variable created by dichotomizing the score into the subscale’s upper quartile. Cronbach’s alpha was 0.91 in our sample.

The dependent variable *other mental health problems* was assessed using the Brief Symptom Inventory (BSI-53), measuring the psychological symptom pattern of the respondents [34,35]. The introductory instruction was: “How much has the following symptoms problem distressed or bothered you during the past month?” It was followed by 53 items rated on a five-point scale of distress with possible answers ranging from “not at all” (0) to “extremely” (4). The BSI was scored and profiled in terms of nine subscales—that is, primary symptom dimensions (somatization, obsessive compulsive, interpersonal sensitivity, depression, anxiety, hostility, phobic anxiety, paranoid ideation and psychoticism) and the Global Severity Index (GSI) measuring the overall psychological distress level. In the main analyses, individual dimensions were assessed as binary variables created by dichotomizing the score into the subscale’s upper quartile or below. For the purpose of sensitivity analyses, the mean scores of the subscales were used. Cronbach’s alpha for the GSI was 0.97 in our sample.

The background characteristics gender, age and other basic sociodemographic characteristics were obtained by the questionnaire.

### 2.3. Statistical Analyses

First, the background characteristics of the sample were described. In the next step, the associations of different R/S models (see Figure 1) with attachment anxiety were assessed using a binary logistic regression model adjusted for gender, age and educational status. Subsequently, the procedure was repeated for the associations of R/S models with nine BSI subscales and BSI GSI. Each independent variable was tested in a separate model. As a sensitivity analysis, all steps were repeated using linear regression models; first univariately and in the second step, multivariately, with adjustment for gender, age and educational status. All analyses were performed using the statistical software package IBM SPSS version 25 (IBM, New York, USA).

## 3. Results

### 3.1. Description of the Population

The background characteristics of the sample are presented in Table 1. The sample is a representative sample of the Czech population aged 15 years and over (mean age 46.4, SD = 17.4; 95% confidence interval 45.60–47.21; 48.7% men).

Of the whole sample, 60 respondents reported some kind of conversion experience. The factors that contributed to it were mostly mixed. The highest prevalence showed a spiritual experience (75.0%), the example of other people (71.7%), and a difficult life situation (70.0%).

### 3.2. Anxiety in Close Relationships

Table 2 shows the associations of various R/S models with anxiety in close relationships. Model 2A shows that compared to stable non-religious, unstable non-religious respondents were more likely to report higher anxiety in close relationships with an odds ratio (OR) of 1.31 (95% confidence interval 1.02–1.69, *p* < 0.05). This also held for converts, but only those who perceived God as distant (OR = 2.59 (1.30–5.16), *p* < 0.01) (Model 3).

### 3.3. Other Mental Health Problems

Table 3 shows the results of associations of various R/S models with BSI. The pattern of results differed in various models depending on the way in which the respondents were categorised. While religious and non-religious respondents (Model 1) differed in only two BSI dimensions—similar to respondents in Model 2B—further division and combination of categories revealed significant differences among subgroups among religious as well as non-religious respondents.

Model 2A showed that compared to stable non-religious respondents, those that were unstable and non-religious were more likely to report worse MH, with BSI GSI OR = 1.55 (1.19–2.02, *p* < 0.01) and higher risks of elevated scores in seven of the nine specific BSI dimensions. Converts scored higher on BSI GSI (OR = 2.70 (1.56–4.69), *p* < 0.001) and all nine BSI dimensions. Stable religious respondents scored higher on BSI GSI (OR = 1.34 (1.01–1.76), *p* < 0.05) and two BSI dimensions.

Model 3 revealed further differences within the religious group, showing that converts who perceived God as distant demonstrated the worst MH. They scored higher on BSI GSI (OR = 4.08 (2.03–8.22), *p* < 0.001), as well as all BSI dimensions. In contrast, converts who perceived God as close differed in only three BSI dimensions, with no significant differences in the BSI GSI. Similarly, within the stable religious group, only the religious who perceived God as distant scored higher on BSI GSI (OR = 1.36 (1.01–1.85), *p* < 0.05) and two BSI dimensions, while the stable religious respondents who perceived God as close did not show any statistical difference from the stable non-religious group.

The results of the sensitivity analysis using linear regression models are presented in the Supplementary Materials Tables S1 and S2. In general, the patterns of the results regarding significant associations were in line with the pattern of the results presented in Table 2 and Table 3.

## 4. Discussion

The aim of this study was to contribute to our understanding of the heterogeneity of findings in the associations of religiosity with MH. We found that different approaches to assessing religiosity (i.e., a different categorisation of respondents based on other related concepts) led to different findings. Unstable non-religious respondents and converts who perceived God as distant were more likely to experience anxiety in close relationships. Furthermore, we found higher risks of worse MH for unstable non-religious respondents, for converts who perceived God as distant and for stable religious respondents who perceived God as distant.

We found that the unstable religious respondents (i.e., the non-believers who reported that their attitude could change in case of need and distress) were more likely to report a higher attachment anxiety compared with stable non-religious respondents. Therefore, their religious instability could already be a symptom of worse MH, associated with the inclination to search for some external source of strength and support in times of need. This religious instability may subsequently manifest itself in a shift towards religion. This agrees with the findings of other authors who mention attachment insecurity as one of the factors in the conversion process [20]. Moreover, these respondents showed higher risks of seven BSI symptoms and the BSI GSI score compared with the stable non-religious respondents. These findings are in line with the study of Zinnbauer and Pargament [29], in which the convert group showed more pre-conversion perceived stress and a greater sense of personal inadequacy and limitation before the conversion. Our results could also be supported by studies which reported more adverse health outcomes among respondents who were inconsistent in their religiosity and spirituality [36]—that is, spiritual non-believers [37,38]. This suggests that religious instability related to a more general instability in attachment may provide some explanation for the heterogeneity in the findings regarding S/R and mental health.

We also found that converts who perceived God as distant were more likely to experience anxiety in close relationships. These findings further support the idea of the association between one’s attachment style and religious conversion [39]. Moreover, these respondents showed approximately a four-times higher risk of worse MH than stable non-religious respondents. These findings contrast with those of other authors, who, for example, associated conversion with a decrease in symptoms of depression and hopelessness [40] and positive personality changes [41]. Nevertheless, as 70% of our respondents reported that a difficult life situation played an important role in their conversion, our results are consistent with the idea of conversion as a search for security in a difficult time [20]. In addition, in secular countries, a stronger impulse (e.g., a worse psychological condition) might be needed for conversion. The fact that this concerns only the converts who perceive God as distant suggests that the subgroup with better MH may represent respondents with a secure attachment style, while the other respondents have an insecure attachment style, as some other authors have also suggested [42]. Alternatively, spiritual experience is often associated not only with the perceived closeness to God, but also feelings of peace, happiness and meaning of life [43]. This may in turn lead to better mental health.

### 4.1. Strengths and Limitations

This study has several important strengths. The most important is that it explores various explanations of deviating findings on R/S and MH and points to possible sources of the heterogeneity. It is also the first study that uses a representative sample to assess the associations of various R/S models with MH in the secular environment of the Czech Republic. To the best of our knowledge, it is also the first study which categories the respondents according to the stability of religious attitudes, and therefore offers another point of view. A limitation of our study is its cross-sectional design, which does not allow us to make conclusions on causality; there was also a small number of converts compared to the other groups in the study. However, the latter is a natural consequence of assessing a representative sample in a secular environment. Another limitation is that we did not consider all genders, but only men and women. However, as the gender differences were not the main focus of this study, we do not presume that this influenced the validity of the present study. The last limitation might be an information bias, as our data were based on the self-reports of respondents, which can be influenced by social desirability. This is hard to avoid given the subjective nature of the various issues.

### 4.2. Implications

Our findings show that combining measures on religiousness with measures on closeness to God and the stability of religious attitudes could yield outcomes that lead to a different interpretation. The results of various studies on R/S that do not take these factors into account should therefore be interpreted with caution.

We also found that both religious and non-religious respondents represent heterogeneous groups regarding these related characteristics, and that a more comprehensive measurement is needed if research in this area is to have any practical implications (e.g., for psychologists and psychiatrists). Our findings should also be confirmed in further research using a similar methodology, as they are new regarding their approach in a secular setting. Moreover, further research on R/S and health is needed to clarify the causality direction in this area.

Moreover, we found that religious respondents differ in their MH problems depending on their perceived closeness to God. Further research is needed to explore whether the secure attachment to God corresponds solely to the attachment style gained in childhood or through life (correspondence pathway), or whether it could also be gained through an experienced relationship with God (compensation pathway) [19]. In the latter case, one’s image of God could represent a potential target of psychotherapeutic/spiritual intervention.

## 5. Conclusions

Our findings suggest that the heterogeneity of findings in associations of R/S with MH could be due to measurement problems (i.e., not taking into account the heterogeneity of the groups of religious and non-religious people). A different categorisation of respondents regarding their religiosity could possibly lead to outcomes with different interpretations. A shift towards religiosity could be expected in a substantial part of the non-religious respondents in problematic times, which highlights the issues with causality in measuring R/S and MH in a secular country. Our results also show a link with the adult attachment style, which may help in the interpretation of the results. Further research in this area is needed with regard to the clinical applicability of these findings.

## Figures and Tables

**Table 1:** Description of the study population, total and by stability of religiousness.

	Total		Stable Non-Religious		Unstable Non-Religious		Converts		Stable Religious	
	*n*	%	*n*	%	*n*	%	*n*	%	*n*	%
**Gender**										
Male	873	48.6	412	47.2	230	26.3	27	3.1	204	23.4
Female	922	51.4	329	35.7	293	31.8	33	3.6	267	29.0
**Age**										
15–29 years old	409	22.8	196	47.9	127	31.1	8	2.0	78	19.1
30–44 years old	448	25.0	206	46.0	137	30.6	14	3.1	91	20.3
45–59 years old	441	24.6	183	41.5	118	26.8	17	3.9	123	27.9
60–90 years old	497	27.7	156	31.4	141	28.4	21	4.2	179	36.0
**Living arrangement**										
With husband/wife	917	51.1	362	39.5	250	27.3	35	3.8	270	29.4
With unmarried mate	350	19.5	158	45.1	125	35.7	6	1.7	61	17.4
Alone	353	19.7	144	40.8	93	26.3	13	3.7	103	29.2
With parents/siblings	175	9.7	77	44.0	55	31.4	6	3.4	37	21.2
**Marital status**										
Single/Divorced/Widow(er)	729	40.6	302	116.0	228	89.0	23	11.9	176	83.1
Married/Partner relationship	1066	59.4	439	91.4	295	57.7	37	5.2	295	45.7
**Highest education achieved**										
Elementary school	140	7.8	64	45.7	39	27.9	4	2.9	33	23.6
Secondary vocational school	441	24.6	173	39.2	118	26.8	20	4.5	130	29.5
Secondary school with graduation	852	47.5	360	42.3	258	30.3	21	2.5	213	25.0
College	362	20.2	144	39.8	108	29.8	15	4.1	95	26.2
**Economic activity**										
Employee	938	52.3	414	44.1	283	30.2	31	3.3	210	22.4
Self-employed	167	9.3	79	47.3	43	25.7	5	3.0	40	24.0
Household ^a^/Unemployed	83	4.6	38	92.2	18	42.5	3	7.9	24	57.4
Student	178	9.9	80	44.9	59	33.1	2	1.1	37	20.8
Disabled/Old-age pensioner	429	23.9	130	67.1	120	48.5	19	14.4	160	60.0
**Religiosity ^b^**										
Believer, church member	170	9.5	0	0.0	0	0.0	32	18.8	138	81.2
Believer outside the church	361	20.1	0	0.0	0	0.0	28	7.8	333	92.2
Non-believer	1001	55.8	563	56.2	438	43.8	0	0.0	0	0.0
Convinced atheist	263	14.7	178	67.7	85	32.3	0	0.0	0	0.0
Total	1795	100	741	41.3	523	29.1	60	3.3	471	26.2

Note: ^a^ including maternity leave. ^b^ independent of church attendance.

**Table 2:** Associations of different religiosity/spirituality (R/S) models with anxiety in close relationships: results of binary logistic regression with the stable non-religious respondents being the reference category, adjusted for age, gender and education level leading to odds ratios with 95% confidence intervals.

Model		*N*^a^ (%)	Anxiety in Close Relationships OR (95% CI)
1	Non-religious	1264 (70.4)	1
	Religious	531 (29.6)	0.88 (0.69–1.12)
2A	Stable non-religious	741 (41.3)	1 *
	Unstable non-religious	523 (29.1)	1.31 (1.02–1.69) *
	Converts	60 (3.3)	1.70 (0.97–2.97)
	Stable religious	471 (26.2)	0.92 (0.70–1.22)
2B	Non-religious	1264 (70.4)	1
	Religious, who perceive God as distant	374 (20.8)	0.94 (0.72–1.22)
	Religious, who perceive God as close	157 (8.7)	0.76 (0.50–1.14)
3	Stable non-religious	741 (41.3)	1 *
	Unstable non-religious	523 (29.1)	1.31 (1.02–1.69) *
	Converts, who perceive God as distant	35 (1.9)	2.59 (1.30–5.16) **
	Converts, who perceive God as close	25 (1.4)	0.82 (0.30–2.22)
	Stable religious, who perceive God as distant	339 (18.9)	0.95 (0.70–1.28)
	Stable religious, who perceive God as close	132 (7.4)	0.86 (0.55–1.35)

Notes: * *p* < 0.05, ** *p* < 0.01; Significant associations are highlighted in bold font; ^a^ Total number of respondents in each category.

**Table 3:** Associations of different R/S models with selected Brief Symptom Inventory (BSI) symptoms and the Global Severity Index (GSI): results of binary logistic regression with the stable non-religious respondents being the reference category, adjusted for age, gender and education level leading to odds ratios (OR) with 95% confidence intervals (95% CI).

Model		*N*^a^ (%)	Somatization	Obsessive Compulsive	Interpersonal Sensitivity	Depression	Anxiety
1	Non-religious	1264 (70.4)	**1**	**1**	1	1	1
	Religious	531 (29.6)	1.29 (1.01–1.64) *	1.61 (1.25–2.07) ***	1.08 (0.83–1.39)	1.17 (0.93–1.47)	1.23 (0.98–1.55)
2A	Stable non-religious	741 (41.3)	1 **	1 ***	1 **	1 **	1 ***
	Unstable non-religious	523 (29.1)	1.42 (1.07–1.88) **	1.28 (0.94–1.73)	1.33 (1.00–1.78) *	1.29 (0.99–1.67)	1.63 (1.26–2.12) **
	Converts	60 (3.3)	2.51 (1.42–4.43) ***	4.42 (2.54–7.68) ***	2.68 (1.52–4.72) **	2.89 (1.68–4.97) ***	4.26 (2.48–7.33) ***
	Stable religious	471 (26.2)	1.40 (1.05–1.87) *	1.55 (1.15–2.11) **	1.09 (0.80–1.48)	1.16 (0.89–1.53)	1.32 (1.00–1.74)
2B	Non-religious	1264 (70.4)	1 **	1 **	1	1	1
	Religious, who perceive God as distant	374 (20.8)	1.52 (1.17–1.99) **	1.67 (1.26–2.21) ***	1.09 (0.82–1.46)	1.21 (0.93–1.56)	1.21 (0.94–1.58)
	Religious, who perceive God as close	157 (8.7)	0.82 (0.54–1.25)	1.47 0.98–2.19)	1.04 (0.68–1.58)	1.09 (0.75–1.58)	1.27 (0.88–1.84)
3	Stable non-religious	741 (41.3)	1 ***	1 ***	1 **	1 **	1 ***
	Unstable non-religious	523 (29.1)	1.42 (1.07–1.88) *	1.28 (0.94–1.73)	1.33 (1.00–1.78) *	1.29 (0.99–1.67)	1.63 (1.26–2.12) **
	Converts, who perceive God as distant	35 (1.9)	4.41 (2.14–9.11) ***	6.33 (3.14–12.78) ***	3.75 (1.86–7.56) ***	4.52 (2.22–9.17) ***	4.87 (2.43–9.78) ***
	Converts, who perceive God as close	25 (1.4)	1.00 (0.36–2.74)	2.60 (1.09–6.19) *	1.55 (0.61–4.00)	1.52 (0.64–3.61)	3.55 (1.58–7.96) **
	Stable religious, who perceive God as distant	339 (18.9)	1.61 (1.18–2.19) **	1.58 (1.14–2.21) **	1.07 (0.76–1.51)	1.17 (0.86–1.58)	1.32 (0.97–1.79)
	Stable religious, who perceive God as close	132 (7.4)	0.96 (0.60–1.53)	1.48 (0.93–2.36)	1.12 (0.70–1.81)	1.16 (0.76–1.78)	1.33 (0.87–2.05)
			**Hostility**	**Phobic Anxiety**	**Paranoid Ideation**	**Psychoticism**	**Global Severity Index**
1	Non-religious		1	1	1	1	1
	Religious		0.96 (0.74–1.24)	1.16 (0.91–1.49)	1.09 (0.85–1.40)	1.04 (0.80–1.35)	1.19 (0.95–1.51)
2A	Stable non-religious		1 *	1 **	1 **	1 **	1 ***
	Unstable non-religious		1.36 (1.03–1.79) *	1.27 (0.96–1.68)	1.34 (1.01–1.76) *	1.49 (1.11–2.00) **	1.55 (1.19–2.02) **
	Converts		1.87 (1.03–3.39) *	2.68 (1.54–4.68) **	2.54 (1.45–4.44) **	2.43 (1.36–4.36) **	2.70 (1.56–4.69) ***
	Stable religious		1.01 (0.74–1.37)	1.16 (0.86–1.55)	1.11 (0.82–1.49)	1.12 (0.82–1.54)	1.34 (1.01–1.76) *
2B	Non-religious		1	1	1	1	1
	Religious, who perceive God as distant		1.12 (0.84–1.50)	1.21 (0.92–1.60)	1.19 (0.90–1.57)	1.10 (0.82–1.48)	1.25 (0.96–1.63)
	Religious, who perceive God as close		0.60 (0.37–0.99) *	1.05 (0.70–1.58)	0.87 (0.57–1.33)	0.90 (0.58–1.40)	1.06 (0.73–1.56)
3	Stable non-religious		1 **	1 *	1 **	1 **	1 ***
	Unstable non-religious		1.36 (1.03–1.79) *	1.27 (0.96–1.68)	1.34 (1.01–1.76) *	1.49 (1.11–2.00) **	1.55 (1.19–2.02) **
	Converts, who perceive God as distant		3.17 (1.56–6.44) **	2.91 (1.44–5.91) ***	4.65 (2.33–9.29) ***	3.91 (1.94–7.91) ***	4.08 (2.03–8.22) ***
	Converts, who perceive God as close		0.64 (0.19–2.18)	2.38 (1.02–5.53) *	0.83 (0.28–2.47)	1.01 (0.34–3.01)	1.44 (0.59–3.52)
	Stable religious, who perceive God as distant		1.14 (0.82–1.59)	1.22 (0.89–1.69)	1.15 (0.83–1.59)	1.13 (0.80–1.61)	1.36 (1.01–1.85) *
	Stable religious, who perceive God as close		0.70 (0.40–1.21)	0.99 (0.62–1.59)	1.02 (0.63–1.64)	1.09 (0.66–1.79)	1.28 (0.83–1.96)

Notes: * *p* < 0.05, ** *p* < 0.01, *** *p* < 0.001; Significant associations are highlighted in bold font; ^a^ Total number of respondents in each category.

## Supplementary Materials

The following are available online at https://www.mdpi.com/1660-4601/17/2/494/s1, Table S1: Associations of different R/S models with anxiety in close relationships: results of linear regression, adjusted for age, gender and education level., Table S2: Associations of different R/S models with selected BSI symptoms and the GSI: results of linear regression, adjusted for age, gender and education level.
