# Supplementary material for: Religiosity and Mental Health: A Contribution to Understanding the Heterogeneity of Research Findings
Source: Int J Environ Res Public Health. 2020 Jan 13;17(2):494. doi: 10.3390/ijerph17020494 (PMC7014109; doi:10.3390/ijerph17020494)
Supplement: Supplementary file 1 [file ijerph-17-00494-s001.pdf]

Supplementary Table 1 Associations of different R/S models with anxiety in close relationships: results of linear regression, adjusted for age, gender and education level.

**Model**		**Anxiety in close relationships**	
		**B**	**Std. Error**
**1**	Non-religious	**1**	
	Religious	**-2.64****	**1.00**
**2A**	Stable non-religious	**1**	
	Unstable non-religious	**3.89*****	1.09
	Converts	2.24	2.55
	Stable religious	-1.38	1.14
**2B**	Non-religious	**1**	
	Religious, who perceive God as distant	-1.29	1.13
	Religious, who perceive God as close	**-5.86*****	1.62
**3**	Stable non-religious	**1**	
	Unstable non-religious	**3.90*****	1.09
	Converts, who perceive God as distant	**8.06***	3.28
	Converts, who perceive God as close	-2.05	4.13
	Stable religious, who perceive God as distant	-0.41	1.26
	Stable religious, who perceive God as close	**-3.87***	1.80

Notes: *p < 0.05, **p < 0.01, ***p < 0.001; Significant associations are highlighted in bold font

Supplementary Table 2 Associations of different R/S models with selected BSI symptoms and the GSI: results of linear regression, adjusted for age, gender and education level.

**Model**		**Somatization**		**Obsessive Compulsive**		**Interpersonal sensitivity**		**Depression**		**Anxiety**	
		**B**	**Std. Error**	**B**	**Std. Error**	**B**	**Std. Error**	**B**	**Std. Error**	**B**	**Std. Error**
1	Non-religious	**1**		**1**		1		1		1	
	Religious	**0.09****	0.03	**0.12*****	0.03	0.06	0.04	0.07	0.04	**0.09****	0.03
2A	Stable non-religious	**1**		**1**		**1**		**1**		**1**	
	Unstable non-religious	**0.07***	0.03	**0.10****	0.03	**0.13****	0.04	0.07	0.04	**0.11****	0.03
	Converts	**0.42****	0.08	**0.52****	0.08	**0.51****	0.09	**0.57****	0.09	**0.60*****	0.08
	Stable religious	**0.08***	0.03	**0.12****	0.04	0.07	0.04	0.04	0.04	0.08*	0.03
2B	Non-religious	**1**		**1**		1		1		1	
	Religious, who perceive God as distant	**0.13****	0.03	**0.14****	0.04	**0.09***	0.04	**0.10***	0.04	**0.10****	0.03
	Religious, who perceive God as close	-0.01	0.05	0.08	0.05	0.003	0.06	0.001	0.06	0.06	0.05
3	Stable non-religious	**1**		**1**		**1**		**1**		**1**	
	Unstable non-religious	**0.07***	0.03	**0.10****	0.03	**0.13****	0.04	0.07	0.04	**0.11****	0.03
	Converts, who perceive God as distant	**0.66****	0.10	**0.74****	0.10	**0.71****	0.12	**0.80****	0.11	**0.79*****	0.10
	Converts, who perceive God as close	0.08	0.12	0.10	0.13	0.20	0.15	0.24	0.14	**0.27***	0.12
	Stable religious, who perceive God as	**0.11****	0.04	**0.13****	0.04	0.08	0.05	0.06	0.04	**0.08***	0.04
	Stable religious, who perceive God as	0.01	0.05	0.11	0.06	0.03	0.07	-0.01	0.06	0.07	0.05

Notes: *p < 0.050. **p < 0.010. ***p < 0.001; Significant associations are highlighted in bold font

Supplementary Table 2 (continuation)

		Hostility		Phobic Anxiety		Paranoid Ideation		Psychoticism		Global Severity Index	
		**B**	**Std. Error**	**B**	**Std. Error**	**B**	**Std. Error**	**B**	**Std. Error**	**B**	**Std. Error**
1	Non-religious	1		1		1		1		1	
	Religious	-0.01	0.03	0.06	0.03	0.03	0.03	**0.07***	0.03	**0.07***	0.03
2A	Stable non-religious	**1**		**1**		**1**		**1**		**1**	
	Unstable non-religious	**0.08***	0.03	**0.09****	0.03	**0.09***	0.04	**0.10***	0.03	**0.09****	0.03
	Converts	**0.30****	0.08	**0.43*****	0.08	**0.42****	0.09	**0.57****	0.08	**0.48*****	0.07
	Stable religious	-0.01	0.04	0.05	0.04	0.03	0.04	0.05	0.04	0.06	0.03
2B	Non-religious	1		1		1		1		**1**	
	Religious, who perceive God as distant	0.03	0.04	**0.09***	0.03	0.07	0.04	**0.09***	0.03	**0.10****	0.03
	Religious, who perceive God as close	**-0.11***	0.05	-0.01	0.05	-0.07	0.06	0.02	0.05	0.002	0.04
3	Stable non-religious	**1**		**1**		**1**		**1**		**1**	
	Unstable non-religious	**0.08***	0.03	**0.09****	0.03	**0.09***	0.04	**0.10****	0.03	**0.09****	0.03
	Converts, who perceive God as distant	**0.60****	0.10	**0.62*****	0.10	**0.71****	0.11	**0.86****	0.10	**0.72*****	0.09
	Converts, who perceive God as close	-0.05	0.13	0.18	0.13	0.06	0.14	0.12	0.12	0.12	0.11
	Stable religious, who perceive God as	0.01	0.04	0.07	0.04	0.05	0.04	0.05	0.04	**0.07***	0.03
	Stable religious, who perceive God as	-0.07	0.06	-0.005	0.06	-0.04	0.06	0.04	0.05	0.02	0.05

Notes: *p < 0.050. **p < 0.010. ***p < 0.001
